# Immune‐check blocking combination multiple cytokines shown curative potential in mice tumor model

**DOI:** 10.1002/cam4.6053

**Published:** 2023-05-18

**Authors:** Hongxia Su, Hui Geng, Linkang Cai, Minjie Xu, Wenpin Xing, Wei Long, Biao Liu, Yankun Li, Binlei Liu

**Affiliations:** ^1^ School of Pharmacy Hubei University of Science and Technologyc Xianning China; ^2^ School of Life Science Huazhong Normal University Wuhan China; ^3^ Wuhan Binhui Biopharmaceutical Co., Ltd. Wuhan China; ^4^ Hubei University of Technology Wuhan China

**Keywords:** GM‐CSF, IL‐12, IL‐2/15, PD‐1v, tumor immunity

## Abstract

**Objective:**

In order to ensure the stable transcription of target genes, we constructed a eukaryotic high expression vector carrying an immune‐check inhibitor PD‐1v and a variety of cytokines, and studied their effects on activating immune response to inhibit tumor growth.

**Methods:**

A novel eukaryotic expression plasmid vector named pT7AMPCE containing T7RNA polymerase, T7 promoter, internal ribosome entry site (IRES), and poly A tailing signal was constructed by T4 DNA ligase, on which homologous recombination was used to clone and construct the vector carrying PD‐1v, IL‐2/15, IL‐12, GM‐CSF, and GFP. In vitro transfection of CT26 cells was performed, and the protein expression of PD‐1v, IL‐12 and GM‐CSF was detected by Western blot and ELISA after 48 h. Mice were subcutaneously inoculated with CT26‐IRFP tumor cells in the rib abdomen, and the tumor tissues were injected with PD‐1v, IL‐2/15, IL‐12, and GM‐CSF recombinant plasmids for treatment during the experimental period. The efficacy of the treatment was evaluated by assay tumor size and survival time of tumor‐bearing mice during the experiment. Expression levels of IFN‐γ, TNF, IL‐4, IL‐2, and IL‐5 in mouse blood were measured using the CBA method. Tumor tissues were extracted and immune cell infiltration in tumor tissues was detected by HE staining and the IHC method.

**Results:**

The recombinant plasmids carrying PD‐1v, IL‐2/15, IL‐12, and GM‐CSF were successfully constructed, and the Western blot and ELISA results showed that PD‐1v, IL‐12, and GM‐CSF were expressed in the supernatant of CT26 cells 48 h after in vitro cell transfection. The combined application of PD‐1v, IL‐2/15, IL‐12, and GM‐CSF recombinant plasmids significantly inhibited tumor growth in mice, and the tumor growth rate was significantly lower than that in the blank control group and GFP plasmid control group (*p* < 0.05). Cytometric bead array data suggested that the combination of PD‐1v and various cytokines can effectively activate immune cells. HE and IHC analysis revealed plenty of immune cell infiltrates in the tumor tissue, and a large proportion of tumor cells showed the necrotic phenotype in the combination treatment group.

**Conclusion:**

The combination of immune check blockade and multiple cytokine therapy can significantly activate the body's immune response and inhibit tumor growth.

## Graphical abstract

The combination of immune check blockade and multiple cytokine therapy can significantly activate the body's immune response and inhibit tumor growth. Our manuscript provides a paradigm for future tumor immunotherapy.

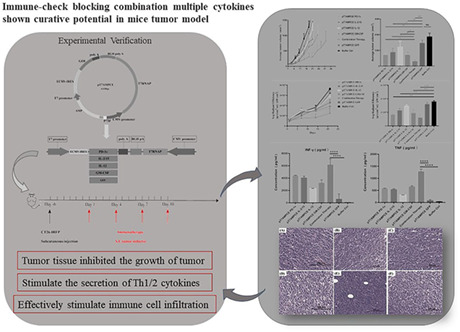

## 1 INTRODUCTION

Tumor microenvironment (TME) is an important site that regulates tumor development, growth, invasion and distant metastasis, and plays a key role in inducing tumor immune escape.^1^, ^2^, ^3^ Many types of tumor cells, such as melanoma, colon cancer, lung cancer, ovarian cancer, head and neck squamous cell carcinoma and glioma, can inhibit the killing function of T cells, increase the apoptosis of tumor‐specific cytotoxic T lymphocyte (CTL), and facilitate tumors to escape from immune surveillance and killing by overexpressing PD‐Ls (including PD‐L1 and PD‐L2), which interact with the negative regulatory receptor programmed death 1 (PD‐1) on the surface of T lymphocytes.^4^, ^5^, ^6^, ^7^ PD‐1 is an important negative regulatory receptor on the surface of T cells after the discovery of CTLA‐4. The binding of PD‐1 and PD‐Ls is intended to prevent excessive activation of T cells and avoid inflammatory damage caused by excessive activation of T cells. However, tumor cells can induce the dysfunction and depletion of infiltrating lymphocytes through high expression of PD‐Ls, so that tumors can escape immune surveillance and killing.^8^, ^9^, ^10^ In recent years, studies have shown that antibody blocking targeting PD‐1/PD‐Ls, namely T‐cell immune check point blocking, cutting off the PD‐1/PD‐Ls signaling pathway, can reverse the dysfunction and depletion of effector T cells and restore the antitumor activity of T cells.^11^, ^12^, ^13^, ^14^

In addition to T lymphocytes, other innate immune cells, including natural killer (NK) cells, NKT cells, macrophages, dendritic cells, mast cells, and neutrophils, also play an important role in controlling tumor growth.^15^ As is known, tumor cells downregulated or lacked MHC class I molecules, and lacked the ability to present tumor‐related antigens, making tumor cells present a low immunogenicity phenotype and thus escape the immune surveillance of the body.^16^, ^17^, ^18^, ^19^ MHC class I molecules expressed on the surface of normal cells are the main molecules to avoid killing attack of self‐NK cells.^20^ Therefore, NK cells recognize tumor cells with low MHC expression, and have a strong anti‐tumor effect in the tumor microenvironment.^21^, ^22^ It was found that, on the one hand, NK cells can migrate to the tumor microenvironment under the effect of IL‐15 and IL‐2, improving the cytotoxicity of NK cells and the recognition of tumor cells.^23^, ^24^, ^25^ On the other hand, NK cells have immunomodulatory potential and can secrete cytokines and chemokines IFN‐γ, TNF‐α, CCL5, XCL1, and granulocyte macrophage colony stimulating factor (GM‐CSF) to facilitate innate and adaptive cellular anti‐tumor responses.^26^, ^27^, ^28^

Attracting immune cells into the tumor microenvironment, as well as the interaction between immune cells, cell relocation, and functional activation greatly affect the anti‐tumor immune response.^29^, ^30^ In early research, people tried to use single cytokines such as IL‐12 and IFN‐γ treatment of tumor, but the effect is not significant. Recent studies have shown that delivering multiple cytokines such as IL‐12, IL‐15, and GM‐CSF with herpes simplex virus type 1 or type 2 as the carrier can effectively mobilize the anti‐tumor immune response of the body, suggesting that the combined application of multiple cytokines is a promising strategy to mobilize the anti‐tumor immune response of the body.^31^, ^32^, ^33^, ^34^

Some studies have shown that although the viral vector has high transfection efficiency, its application is greatly limited due to the potential security risks, such as the occurrence of leukemia induced by some retroviruses, and the limited capacity of the viral vector.^35^ In contrast, nonviral vectors have the advantages of low cost, simple preparation, easy mass production, high safety, and unlimited length of exogenous genes.^36^ Plasmid vector is an attractive in vivo transfection vector in animals that can be injected directly into specific tissues and can achieve effective higher levels of gene expression in vivo. A large number of studies have reported that IRES structures from some viruses help eukaryotic mRNA lacking the 5 ‘cap structure to translate and synthesize proteins.^37^ Also, studies have shown that adding poly(A) to T7 promoter‐initiated transcription units can prolong mRNA life and improve translation efficiency.^38^ In order to improve the efficiency of gene expression, especially the translation of target gene, we used molecular cloning technology to construct a new dual promoter plasmid expression vector pT7AMPCE carrying CMV promoter, T7 RNA polymerase (T7RNAP), BGHpA, T7 promoter, IRES structure and poly(A), aiming to express T7RNAP with CMV promoter, T7RNAP transcribes IRES, target genes and poly(A), and used to carry the immune check point inhibitor PD‐1v and a variety of cytokines. To explore its role in attracting and driving immune cells into tumor tissue and restoring anti‐tumor immune response.

## 2 MATERIALS AND METHODS

### 2.1 Ethics statement

All animal experiments were conducted under the protocol approved by the Hubei Province, P.R. China, Biological Studies Animal Care and Use Committee.

### 2.2 Cell lines and mice

Mouse colon cancer CT26 cells were purchased from the National Biomedical Experimental Cell Repository. CT26 cells stably expressing near‐infrared fluorescent protein (IRFP) (referred to as CT26‐IRFP) were constructed in our laboratory by using the PiggyBac transposon system to transfer the near‐infrared fluorescent protein IRFP 720 into the murine colon cancer cell line CT26 (System Bioscience), and the CT26‐IRFP 720 monoclonal stable cell line was obtained by puromycin screening. CT26 and CT26‐IRFP were cultured in Dulbecco's modified Eagle's medium/Nutrient Mixture F‐12 Ham (DME/F‐12) supplemented with 10% fetal bovine serum at 37°C in a 5% CO_2_ incubator.

Six‐ to eight‐week‐old female BALB/C mice were purchased from the Hubei Provincial Center for Disease Control and Prevention (No. 42000600043578). Each experimental group had five BALB/c mice. Animals were housed at a controlled temperature of 20–25°C, relative humidity of 40–70%, with a 12‐h light/dark cycle interval, and adequate supply of water and food. The study was approved by the Ethics Committee of Hubei University of Science and Technology (No. [2021] HBSTC‐05 (981)). All animal experiments were performed in strict accordance with the requirements of the Hubei Provincial Laboratory Animal Management and Use Committee.

### 2.3 Plasmid construction

pT7AMPCE plasmid construction: Primer pairs were designed to amplify fragment 1 (containing immediate early cytomegalovirus promoter, CMVp in short), fragment 2 (containing T7 RNA polymerase, T7RNApol in short), and fragment 3 (containing bovine growth hormone polyadenosine, BGHpA in short). The templates for fragments 1 and 3 were from pcDNA3 (Invitrogen), and the template for fragment 2 was from T7 phage (Invitrogen). Fragment 4 containing CMVp, T7RNA^pol^, and BGHpA was overlap‐PCR amplified using the mixture of fragments 1–3 as the template and with primer pair. Fragment 5 containing pUC plasmid replication origin and ampicillin resistance gene (ampR) was amplified from pSP73 (Promega) using primer pair. Fragment 4 treated with T4 kinase (NEB) and fragment 5 were ligated with T4 DNA ligase (NEB). The ligation reaction was transformed into Top 10 competent cells to generate plasmid pCMVp‐T7RNA^pol^‐BGHpA followed by sequencing verification. Fragment 6 containing T7 promoter (T7p) and encephalomyocarditis virus internal ribosome entry site (ECMV‐IRES) sequence amplified from pcDNA3‐IRES (in house) using primer pair was then cloned into the pCMVp‐T7RNA^pol^‐BGHpA *SspI* site to generate pT7AMPCE.

Recombinant plasmid construction: PD‐1v, IL‐12, GM‐CSF, and GFP gene templates were obtained from the recombinant HSV2 virus constructed earlier in our laboratory, and the IL‐2/15 gene template was obtained from the pHG52D34.5‐CMV‐IL‐2/15 recombinant plasmid preserved earlier in our laboratory.^18^, ^21^ The primer sequences used for cloning are shown in Table 1. The pT7AMPCE plasmid was digested by *Bgl*II and ligated with the above three gene fragments via ClonExpress II recombinase. All cDNAs inserted into the constructed plasmids were confirmed by DNA sequencing.

**TABLE 1:** Primers in plasmid construction.

Fragment	Primer pair
1	P1 TTAGGGTTAGGCGTTTTGC P2 GCGATGTTAATCGTGTCCATGGTGGCGGCAAGCTTGATATCAATTTCGATAAGCCAGTAAG
2	P3 CTTACTGGCTTATCGAAATTGATATCAAGCTTGCCGCCACCATGGACACGATTAACATCGC P4 GTCGAGGCTGATCAGCGAGCTCTAGGATATCTTACGCGAACGCGAAGTCCG
3	P5 CGGACTTCGCGTTCGCGTAAGATATCCTAGAGCTCGCTGATCAGCCTCGAC P6 CTCAGAAGCCATAGAGCC
4	P7 CTTCGCGATGTACGGGCCAGATATACGCGTTGACATTGATTA P8 CAGGATCCTCCCCAGCATGCCTGCTATTGTCTTC
5	P9 TAACCTGCATTAATGAATCG P10 ATAATATTGAAAAAGGAAGAGTA
6	P11 AATAATACGACTCACTATAGGGCTCGAGGCCCCTCTCCCTCCCCCCCCCCTAA P12 TTTTTTTTTTTTTTTTTTTTTTTTTTAGATCTCTCGAGTGTGGCCATATTATCATCGTG
7	P13 TCAAAAAGATCTATGGTGAGCAAGGGCGAGGA P14 TCAAAAAGATCTTTACTTGTACAGCTCGTCCA
PD‐1v	P15 TGGCCACACTCGAGAGATCTGATATCATGCAGATCCCCCAGGCCCC P16 TTTTTTTTTTTTTTAGATCTATGCATTTACTTGCCGGGGCTCAGGCTC
IL‐2/15	P17 TATGGCCACACTCGAGAGATCTATGTACCGGATGCAGCTGCTGAGCT P18 TTTTTTTTTTTTTTAGATCTTCAGCTGAAGATCCAGCTCTGCAGGATG
IL‐12	P19 TGGCCACACTCGAGAGATCTATGTGTCCTCAGAAGCTAACCATCTC P20 TTTTTTTTATGCATCTCGAGTCAGGCGGAGCTCAGATAGCC
GM‐CSF	P21 TGGCCACACTCGAGAGATCTATGTGGCTGCAGAATTTACTTTTCCTGGG P22 TTTTTTTTTTTTTTAGATCTTCATTTTTGGCCTGGTTTTTTGCATTCAAA
GFP	P23 TGGCCACACTCGAGAGATCTATGGTGAGCAAGGGC P24 TTTTTTTTTTTTTTAGATCTTTACTTGTACAGCTCGTCC

### 2.4 Plasmid purification

The plasmids were extracted by conventional alkaline lysis and selectively purified by the spermine method. The endotoxin content was detected by the LAL method below 5 EU/Hg. The purity and conformation of the plasmids were detected by agarose electrophoresis, and the plasmid concentration was quantified and the purity was calculated by a UV spectrophotometer (OD260/OD280). The purity of the plasmids used was greater than 98%.

### 2.5 Cell transfection

The CT26 cells were inoculated in 6‐well plates at 2 × 10^5^ cells/well. When the cell confluence reached 60–70% confluence, DMEM‐F/12 fresh medium without BSA was added into cells. Using lipofectamine 3000 transfection reagent, 3 μg of PD‐1v, IL‐12, and GM‐CSF recombinant plasmids were mixed with lipofectamine 3000 transfection reagent in equal volumes, the mixture was added slowly along the well wall, shaken gently, and incubated at 37°C with 5% CO_2_, and GFP recombinant plasmid was used as positive control in this experiment. After 48 h, the cell supernatant was collected and the expression of the target protein was detected by Western blot and ELISA.

### 2.6 CT26‐IRFP tumor model

Five BALB/c mice were inoculated subcutaneously with 2 × 10^5^ CT26‐IRFP cells (in 100 μL physiological saline) in each group on the right flank. When the tumors grew to an average diameter of 60–80 mm^3^, 80 μg of four different plasmids, including PD‐1v, IL‐2/15, IL‐12, and GM‐CSF, was injected intra‐tumorally into each mouse in the single treatment group, 80 μg of the four plasmid mixtures was injected into the combination treatment group, 100 μg of GFP recombinant plasmid was injected into the empty vector control group, and 100 μL of 0.9% NaCl was injected into the blank control group. Gene delivery was conducted every 3 days for four times in all. Tumor diameter was measured by a Vernier caliper every other day from Day 6. Tumor volume was calculated according to the formula: [(larger axis) × (smaller axis)^2^×0.5]. When the tumor volume reached 1500 mm^3^, tumor volume measurement was stopped and the mice were euthanized to avoid unnecessary suffering.

### 2.7 In vivo animal imaging detection

The mice were anesthetized with isoflurane and then grouped into small animal live imaging system chambers to collect CT26‐IRFP signal intensity. The CT26 fluorescence signal intensity was measured every 7 days and continued until the end of the experiment.

### 2.8 Detection cytokine levels in blood

On Day 14, blood was collected from the orbital vein and serum was separated. Cytometric bead array (CBA) was used to detect the levels of various cytokines in the serum of mice. After the CBA reagent was placed at room temperature for 30 min, the trapping microspheres of IL‐2, IL‐4, IL‐5, IFN‐γ, and TNF were mixed. In brief, 50 μL of capture microspheres, 50 μL of serum to be tested, and 50 μL of PE‐labeled fluorescent antibody were added to each FACS tube. Each tube was washed with 1 mL of washing solution, centrifuged at 200*g* for 5 min, and the supernatant was carefully aspirated. Three hundred microliters of washing solution was added to each tube to resuspend the cells, then analyzed on a NovoCyte Flow Cytometer by using NovoExpress software (Agilent). The obtained data were used to calculate the content of each cytokine in the samples by CBA software FCAPArrayv3.

### 2.9 HE staining and immunohistochemistry

The tumor tissues were surgically excised, fixed overnight in 4% paraformaldehyde, dehydrated, paraffin‐embedded, and cut into 4‐ to 5‐μm‐thick sections. The procedure of immunohistochemical staining was as follows: paraffin sections were routinely dewaxed, rehydrated, and incubated with 3% H_2_O_2_ at room temperature for 5–10 min to eliminate endogenous peroxidase activity. The tissue to be stained was circled with a histochemical oil pen, sealed with 10% FBS for 1 h at room temperature, added primary antibody to be tested, incubated for 2 h at room temperature, washed with PBS, added HRP‐labeled rabbit anti‐mouse IgG antibody, washed with PBS for 1 h at room temperature, developed with DAB, counterstained with nuclei, and dehydrated and sealed.

### 2.10 Statistics

All analyses were done using GraphPad Prism 8 (GraphPad Software). Unless otherwise stated, quantitative data are exhibited as mean ± SD. Statistical data were analyzed by Student's *t*‐test. Mice survival was shown using Kaplan–Meier survival curve. Differences were considered significant at *p* < 0.05 (*p < 0.05; **p < 0.01; ***p < 0.001; ****p < 0.0001); NS, not significant. All data were tested for normality before statistical analysis (GraphPad).

## 3 RESULTS

### 3.1 Construction of expression vector

**FIGURE 1:**
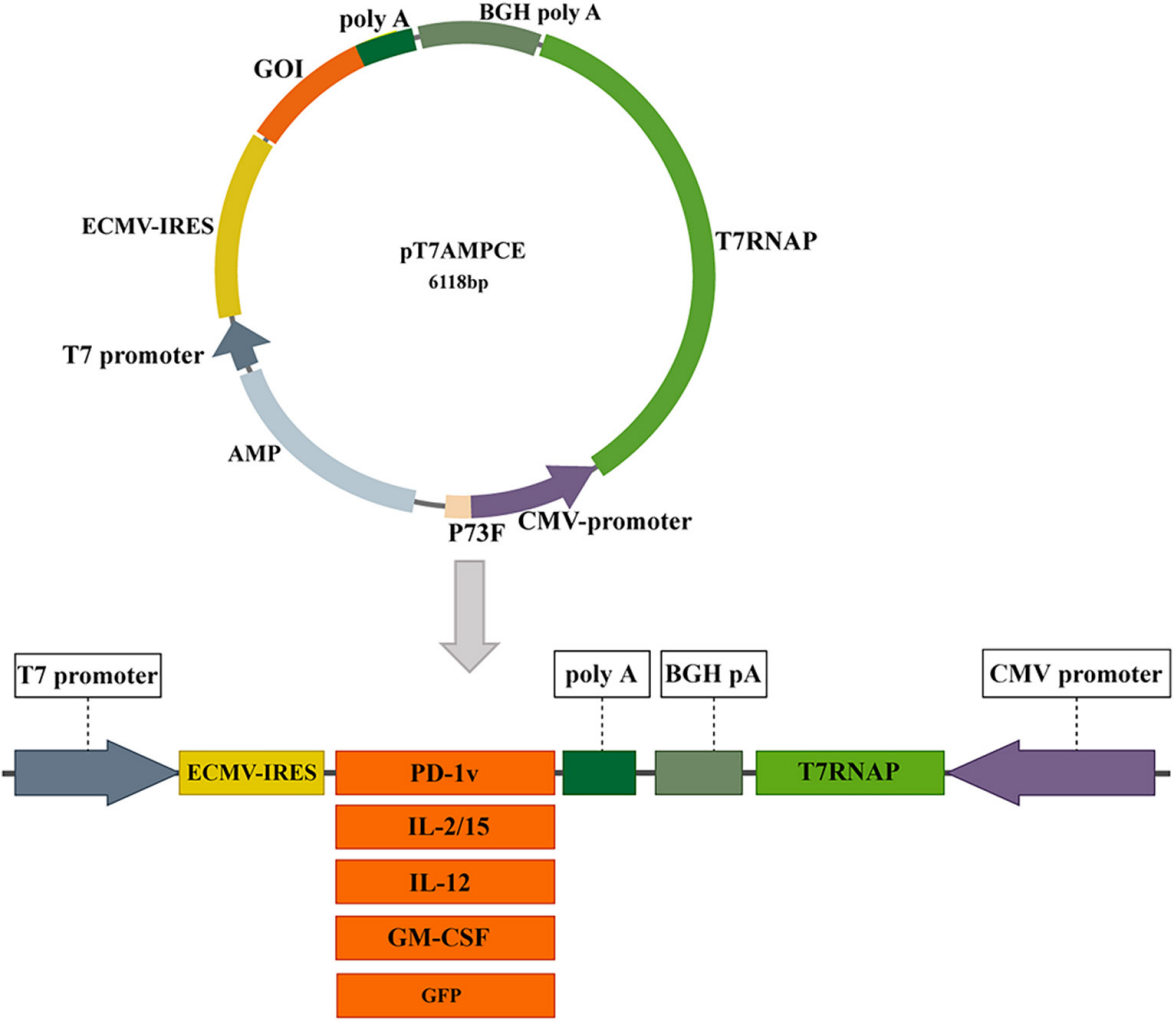
CMV promoter, T7 RNA polymerase (T7RNAP), bovine growth hormone polyadenosine signal (BGH polyA), gene of interest (GOI), T7 promoter, encephalomyocarditis virus internal ribosome entry site (ECMV‐IRES), and ampicillin antibody gene (AMP). Schematic diagram of PD‐1v, IL2/15, IL12, GM‐CSF, and GFP recombinant plasmids constructed from pT7AMPCE as the backbone.

A new plasmid expression system vector carrying CMV promoter, T7 RNA polymerase (T7RNAP), bovine growth hormone polyadenosine signal (BGHpA), internal ribosome entry site (IRES), T7 promoter, and Poly A was constructed using the T4 DNA ligase cloning method. It was named pT7AMPCE, and the construction process is shown in Figure 1. With pT7AMPCE as the backbone, the cDNA of PD‐1v, IL‐2/15, IL‐12, GM‐CSF, and GFP was inserted between IRES and poly A sequences. There is a *Bgl*II restriction site at both ends of the target gene insertion for ease of digestion identification. The five recombinant plasmids obtained were confirmed by DNA sequencing to be consistent with the sequence published by GenBank, indicating that the plasmid construction was correct.

### 3.2 Transgene expression in vitro

**FIGURE 2:**
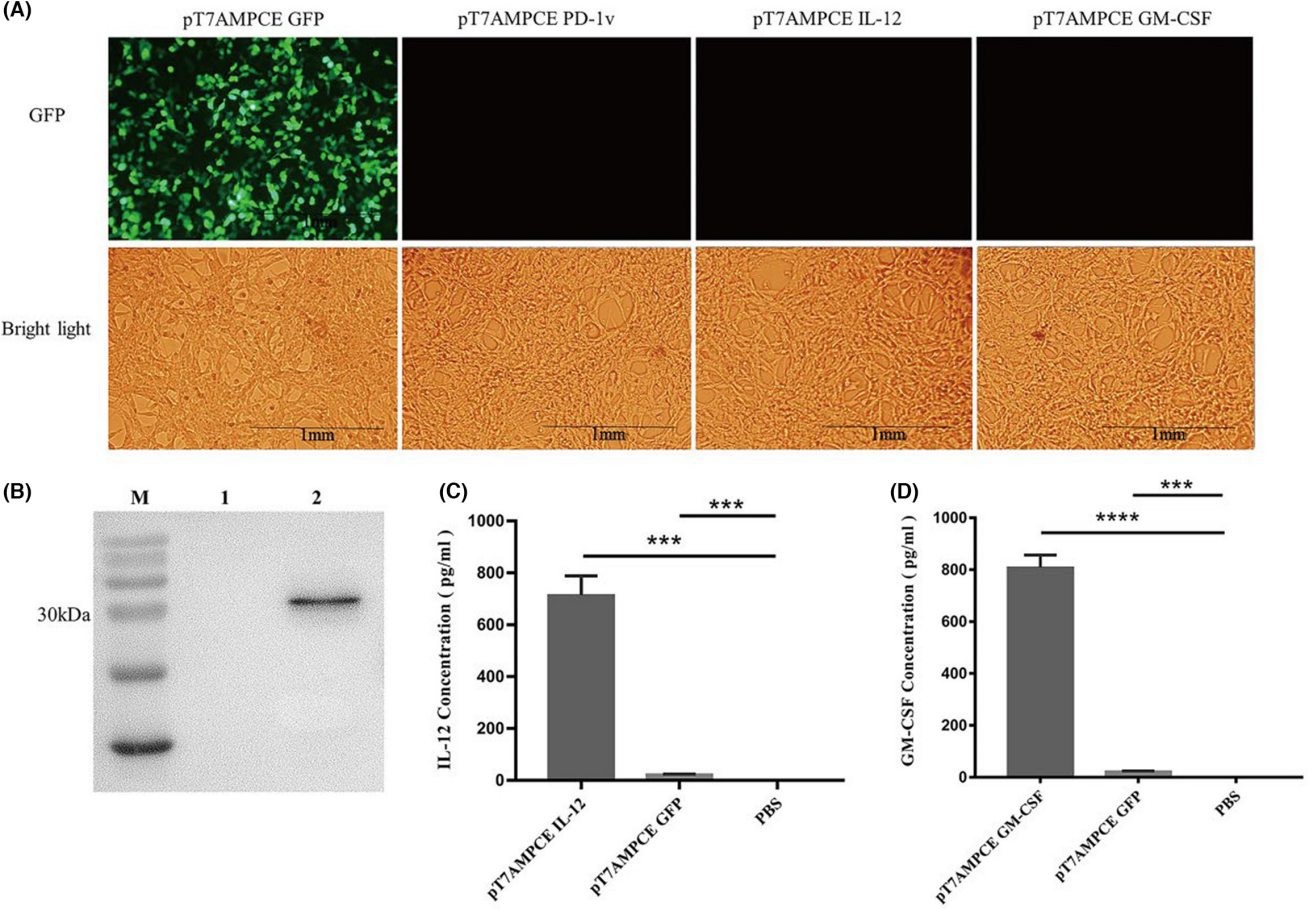
Detection of target protein expression in vitro. (A) Fluorescence detection of CT26 cells after transfection with four plasmids (pT7AMPCE GFP/PD‐1v/IL‐12/GM‐CSF). (B) The expression of PD‐1v (about 33 kDa) was detected by Western blot. Lane 1: supernatant of CT26 cells transfected with GFP plasmid; Lane 2: supernatant of CT26 cells transfected with PD‐1v plasmid. (C) The expression of IL‐12 in CT26 cell supernatant was detected by ELISA. (D) The expression of GM‐CSF in CT26 cell supernatant was detected by ELISA. *p < 0.05; **p < 0.01; ***p < 0.001; ****p < 0.0001.

The recombinant plasmids described above were transfected into CT26 cells, and the supernatant of CT26 cells was collected 48 h later to detect the expression of the target protein. In the in vitro transfection experiment, GFP recombinant plasmid was used as the positive control of transfection. Fluorescence microscopy showed that many cells expressed green fluorescent protein after 48 h transfection with GFP recombinant plasmid (Figure 2A). The expression of PD‐1v was detected by Western blot, and the expressions of GM‐CSF and IL‐12 were detected by ELISA. Western blot results showed a band around 30 kDa in the CT26 cell supernatant after 48 h transfection with PD‐1v recombinant plasmid, which was consistent with the expected molecular weight of PD‐1v (Figure 2B). ELISA detection showed that the concentration of IL‐12 reached 600 pg/mL in the supernatant of CT26 cells transfected with the IL‐12 recombinant plasmid (Figure 2C). The concentration of GM‐CSF reached 700 pg/mL in the supernatant of CT26 cells transfected with the GM‐CSF recombinant plasmid (Figure 2D). The above results suggest that the new pT7AMPCE vector can effectively express the target protein.

### 3.3 Local application of PD‐1v and various cytokines in tumor tissue inhibited tumor growth

**FIGURE 3:**
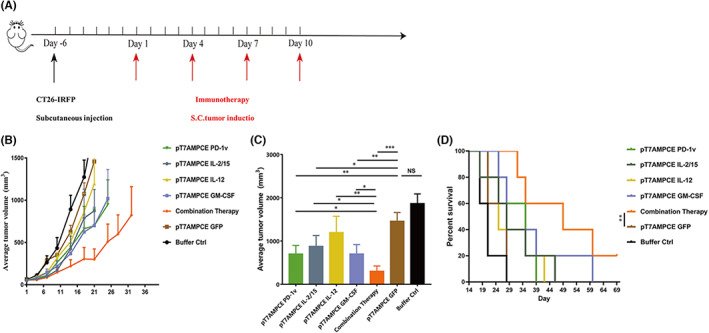
The therapeutic effect of PD‐1v combined with various cytokines in the CT26‐IRFP model. (*n* = 5). (A) Schematic diagram of the time points for tumor cell implantation and plasmid injection in mice. (B) The 21‐day mouse tumor volume growth curve for four single plasmid groups, one combined group, and two control groups. Data are expressed as mean ± SD. The seven groups included pT7AMPCE PD‐1v, IL‐2/15, IL‐12, GM‐CSF, the mixture of the above four plasmids of equal volume, PBS (buffer control), and pT7AMPCE GFP (plasmid control). The first injection time was designated as Day 1. On Days 1, 4, 7, and 10, seven groups of experimental mice were treated intratumorally, with a volume of 80 μL/pc. (C) The tumor volume of mice in single plasmid groups, combined group, and control groups on Day 21 after treatment. Quantitative data are exhibited as mean ± SD. Statistical data were analyzed by Student's *t*‐test. Differences were considered significant at *p* < 0.05 (*p < 0.05; **p < 0.01; ***p < 0.001; ****p < 0.0001); NS, not significant. (D) The survival rate of mice was analyzed by the Kaplan–Meier method until 69 days of continuous observation (**p < 0.01).

In order to observe the effect of PD‐1v and the combined application of various cytokines, CT26‐IRFP cells were subcutaneously inoculated into the right flank of mice to establish a transplanted tumor model. When the average tumor volume reached 60–80 mm^3^, plasmids PD1‐v, IL‐2/15, IL‐12, and GM‐CSF were inoculated into the tumor, four times every 3 days (Figure 3A). The continuous observation for 21 days was performed. Compared with the single‐plasmid treatment group, the vehicle control group (pT7AMPCE GFP), and the solvent control group, the growth trend of tumor in the combined treatment group was much more flat (Figure 3B). Statistical analysis of the tumor volume on Day 21 showed that the tumor volume of the combined treatment group was significantly smaller than that of the PD‐1v and GM‐CSF single plasmid treatment groups (Figure 3C), while the IL‐12 plasmid groups did not show significant therapeutic effect compared with the vehicle control group (Figure 3C). The survival rate of tumor‐bearing mice for 69 consecutive days showed that all the mice in the solvent control group and the vehicle control group died in 29 days. PD‐1v, IL‐12, and IL‐2/15 single plasmid treatment groups showed the mortality rate teached 100% at 39 days, 42 days, and 46 days, respectively. The mortality rate of 60 days after GM‐CSF treatment was 100%, while the survival rate of 69 days in the combined treatment group was 20%, which was significantly different from the control group and the single particle control group (*p* = 0.0019, Figure 3D).

### 3.4 Comparison of fluorescence signal intensity of tumor IRFP gene expression in vivo

**FIGURE 4:**
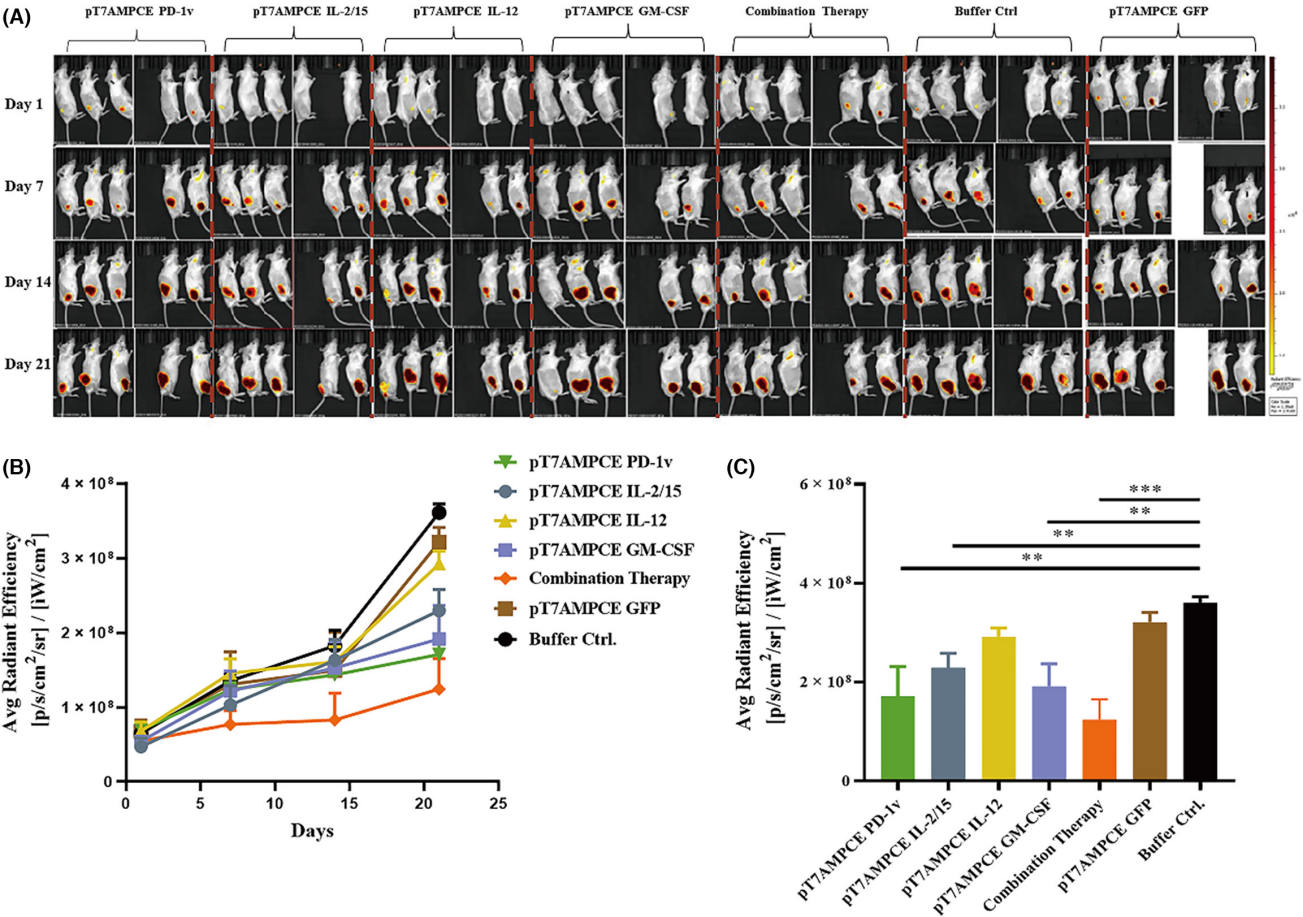
Detection of the fluorescence signal intensity of CT26‐IRFP expressed in vivo (*n* = 5). (A) Fluorescence signal map of IRFP expression in vivo of CT26‐IRFP tumor‐bearing mice treated with different cytokine plasmids. (B) The fluorescence signal trend of tumor IRFP gene expression in vivo of mice in the single plasmid group, combined group, and control group at Day 21. Data are expressed as mean ± SD. (C) Fluorescence signal intensity of mouse tumor IRFP gene expression in vivo of mice in the single plasmid group, combination group, and control group at Day 21 of treatment. Quantitative data are exhibited as mean ± SD. Statistical data were analyzed by Student's *t*‐test. Differences were considered significant at *p* < 0.05 (*p < 0.05, **p < 0.01, ***p < 0.001, ****p < 0.0001).

Small animal biopsy allows for long and staged, noninvasive quantitative detection of tumors in mice, and most critically, small animal biopsy allows for more accurate and intuitive results. Because CT26‐IRFP cells carry fluorescence signal, we used small animal imaging system to observe the changes in fluorescence signal intensity in different treatment groups to indicate the growth of CT26 cells in vivo. Compared with the single plasmid treatment group, vehicle control group, and buffer control group (Buffer Ctrl), the IRFP fluorescence signal intensity of CT26 tumor cells in the combined treatment group increased slowly (Figure 4A, B). The analysis of IRFP fluorescence signal intensity of CT26 tumor cells in each group on Day 21 showed that the fluorescence signal intensity of the combined treatment group was lower than that of plasmid treatment (Figure 4C). However, there was no statistically significant difference between IL‐12 and the vector control group in relation to tumor IRFP gene fluorescence signal intensity (Figure 4C). The above data indicate that the combined therapy has a better anti‐tumor effect than the single‐plasmid therapy.

### 3.5 Combined application of multiple plasmids can effectively stimulate the secretion of Th1 and Th2 cytokines

**FIGURE 5:**
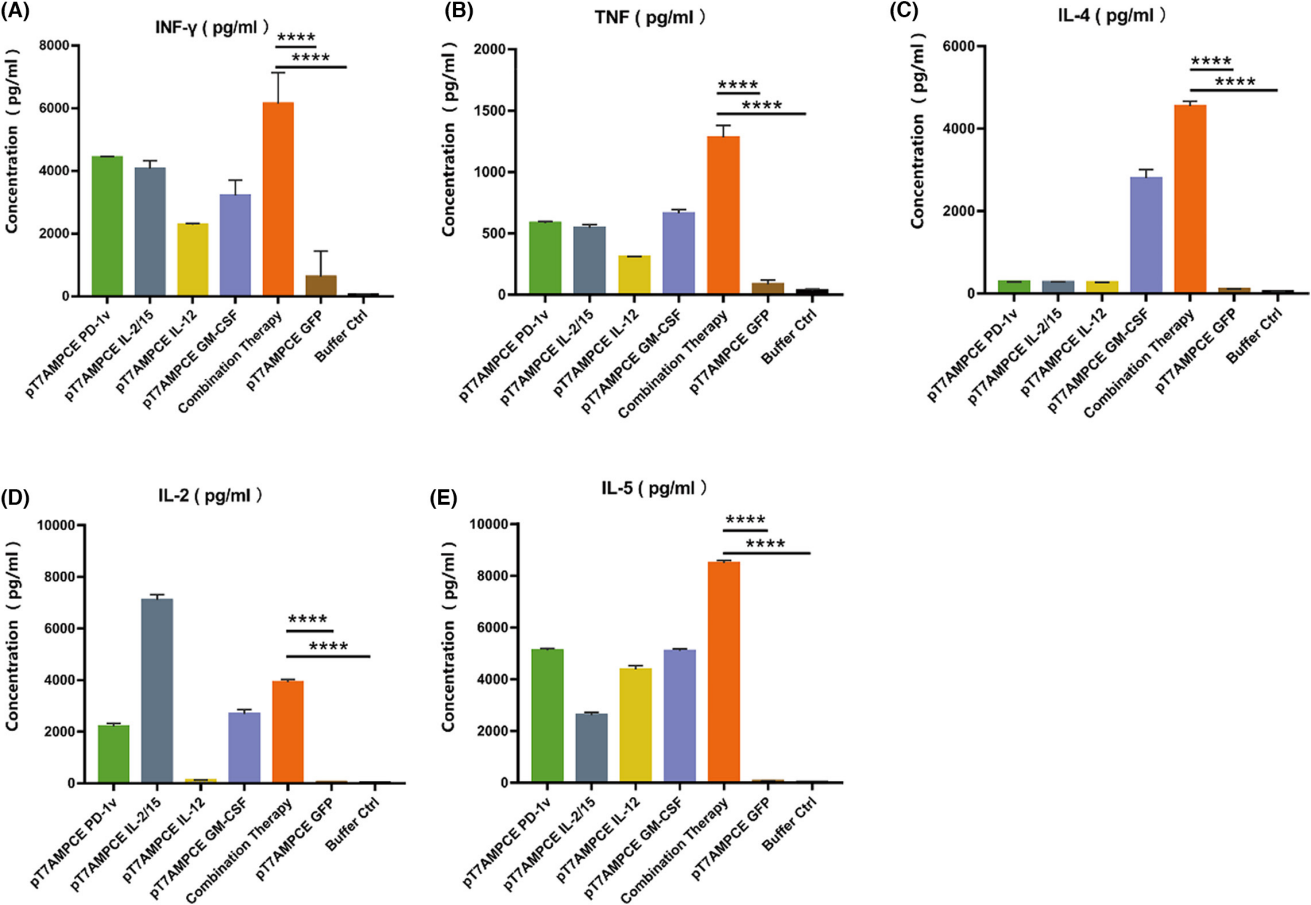
Detection of Th1 and Th2 cytokines in peripheral blood of mice by CBA flow cytometry. Data were analyzed with FlowJo VX software and FCAP Array v3.0 (*n* = 3 biological replicates per experiment; mean ± SD is depicted; unpaired, two‐tailed *t*‐test, *p < 0.05, **p < 0.01, ***p < 0.001, ****p < 0.0001). Secretion level of (B) TNF, (C) IL‐4, (D) IL‐2, and (E) IL‐5.

On Day 14 of treatment, CBA flow cytometry was used to analyze the secretion levels of Th1 and Th2 cytokines in the peripheral blood of mice in each experimental group. The results showed that there was no significant difference in IL‐2 secretion in the IL‐12 treatment group compared with the empty vector control group and the buffer control group (Buffer Ctrl) (Figure 5D). The ability to secrete cytokines IFN‐γ, TNF, and IL‐5 in the single treatment group was increased to a certain extent (Figure 5A, B, E). Compared with the IL‐12 treatment group, the IL‐2 secretion level in the combination treatment group was lower than that in the IL‐12 treatment group (Figure 5D). Cytokines IFN‐γ, TNF, IL‐4, and IL‐5 were upregulated in the combination treatment group compared with the control group and the plasmid treatment group (Figure 5). Our data suggested that the combination of PD‐1v and various cytokines can effectively activate immune cells.

### 3.6 Combined application of multiple plasmids carrying therapeutic genes can effectively stimulate immune cell infiltration

**FIGURE 6:**
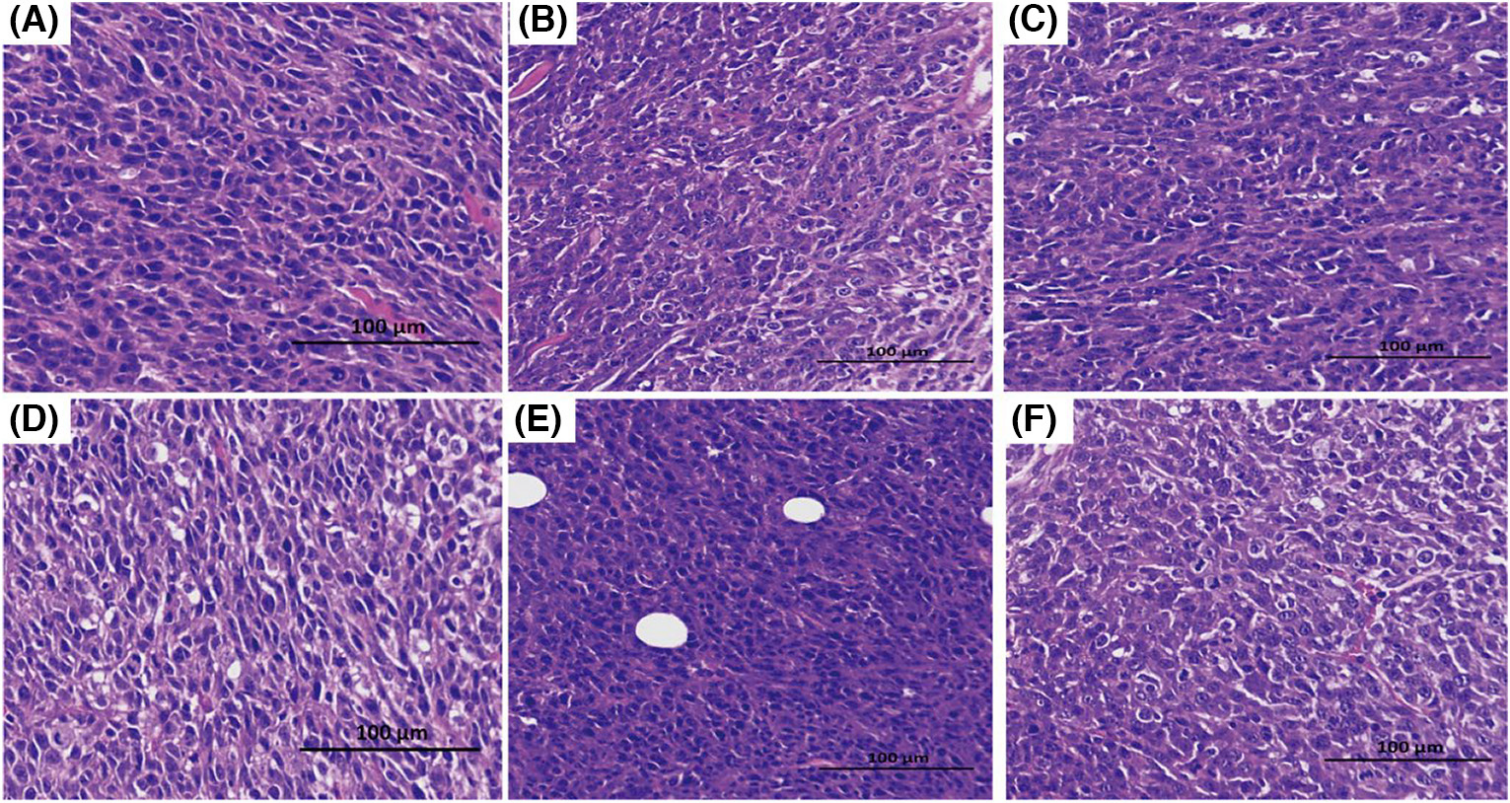
CT26‐IRFP tumor tissue (×200). (A) PD‐1v group; (B) IL 2/15 group; (C) IL‐12 group; (D) GM‐CSF group; (E) combined treatment group; (F) GFP control group: The tumor cells in this group are large and dense, which are typical tumor cells that are not affected.

We also used HE staining to observe the infiltration of immune cells in tumor tissues. The microscopic sections stained with HE showed that immune cell infiltration into the tumor tissue in the combined treatment group was significantly enhanced compared with the control group and the single‐particle treatment groups. In the combined treatment group, there were dense immune cells in the tumor tissue, and some tumor cells showed nucleolus and tumor cell necrosis (Figure 6). It is suggested that the combination of multiple plasmids can effectively attract immune cells into the tumor tissue to play an anti‐tumor role.

**FIGURE 7:**
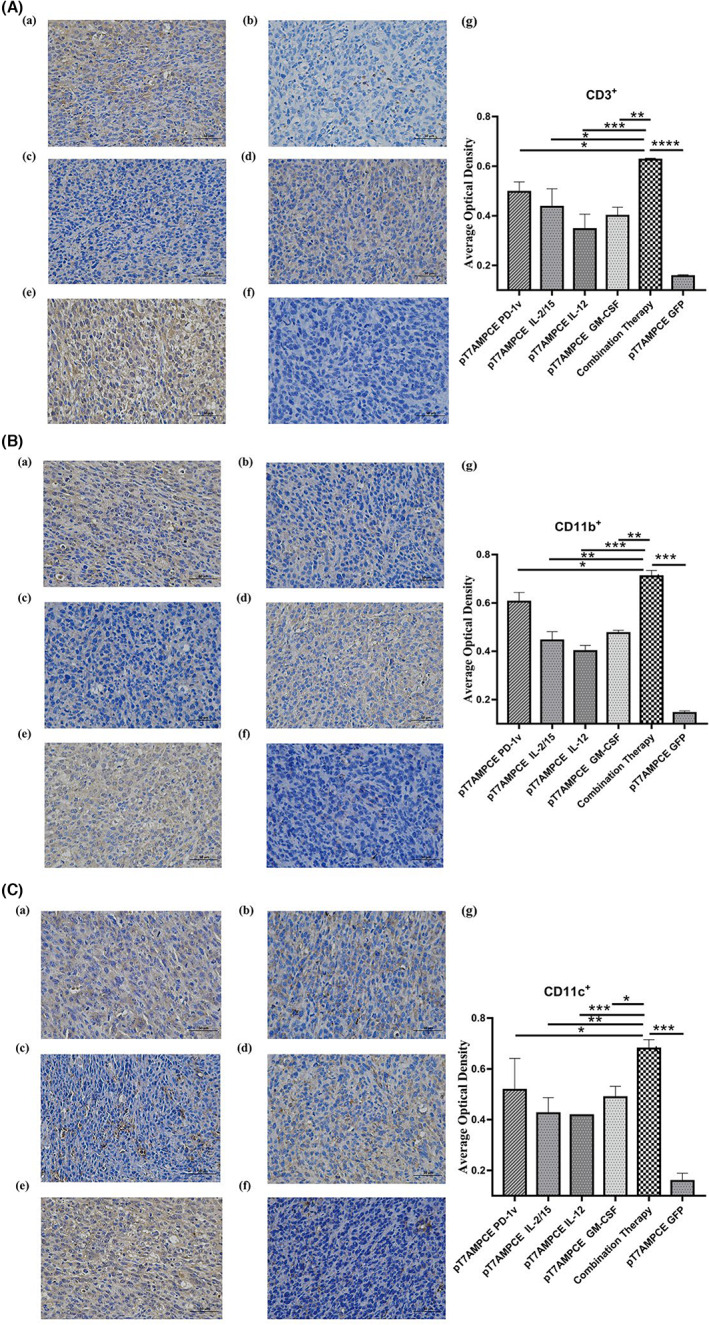
Immunohistochemistry of tumor tissue. The small spots in the figure represent labeled lymphocyte staining (*n* = 5). (A) CD3^+^; (B) CD11b+; (C) CD11c+; (a) pT7AMPCE PD‐1v; (b) pT7AMPCE IL‐2/15; (c) pT7AMPCE IL‐12; (d) pT7AMPCE GM‐CSF; (e) combination therapy; (f) PT7AMPCE GFP control group; (g) statistical analysis chart of stained cells in each experimental group. Quantitative data are exhibited as mean ± SD. Statistical data were analyzed by Student's *t*‐test. Differences were considered significant at *p* < 0.05 (*p < 0.05; **p < 0.01; ***p < 0.001; ****p < 0.0001).

In order to further observe the types of infiltrating cells, we carried out immunohistochemical detection of CD3 (indicating T lymphocytes), CD11b (indicating NK cells), and CD11c (indicating monocytes and macrophages). Figure 7 shows that there are a large number of T lymphocytes, NK cells, monocytes, and macrophages in the tumor tissue of the combined treatment group. It is further suggested that the combination of PD‐1v and various cytokines can effectively enhance the chemoattractant effect on a variety of different immune cells, promote the entry of various immune cells into the tumor tissue, and inhibit the growth of tumor cells.

## 4 DISCUSSION

With more and more in‐depth research on tumor immune escape, tumor immunotherapy has received more attention and has become a treatment method that cannot be ignored in tumor treatment. At present, the most difficult problem of tumor treatment is incompleteness and recurrence, mainly due to tumor immune tolerance and escape, especially the low function of the immune system in the tumor microenvironment. PD‐1 is a negative regulatory receptor on the surface of T cells, which mediates activated T‐lymphocyte depletion by delivering inhibitory signals to activated T cells. Therefore, the therapeutic strategy of blocking the inhibitory signal pathway is particularly important. In previous studies, antibody was used to block the inhibitory signal transmitted by PD‐1, which can enhance the positive immune response against tumors. The anti‐tumor effects achieved in animal experiments and clinical trials have proved this.^1^, ^30^ Unfortunately, the effect of intravenous injection is often systemic, facing serious side effects, and easy to induce autoimmune reaction when treating tumors.^27^, ^38^ Therefore, this paper studies the role of soluble PD‐1 as a local gene therapy agent. PD‐1v recombinant plasmid is used for local injection of tumor tissue, and the expression area is relatively limited, mainly by playing a role in the local role in tumor tissue.

In the process of tumor treatment, it is difficult to achieve good therapeutic effect with single gene therapy. The main reason is that the immune escape mechanism of tumor is multifaceted, especially in the process of treatment, the phenotype of tumor cells in the tumor microenvironment changes, and some escape mechanisms have evolved. Local gene therapy for tumor immune escape mechanism provides a new way to solve this problem. In particular, combined gene therapy can inhibit tumor growth by targeting different tumor escape mechanisms from several aspects. Studies have found that in addition to T lymphocytes, other innate immune cells, including natural killer (NK) cells, NKT cells, macrophages, dendritic cells, mast cells, and neutrophils, also play a key role in controlling tumor growth.^5^ The results of some recent clinical studies combining cytokine gene therapy with different cancer treatments suggest that strategies expressing immunomodulatory genes in solid tumors have some clinical potential. In this study, we used pT7AMPCE as a vector to construct PD‐1v, GM‐CSF, IL‐12, and IL‐2/IL‐15 expression plasmids. In vitro transfection experiments showed that the recombinant plasmid could efficiently express the target gene. After 48 h of transfection, WB detection showed that the PD‐1v expression product formed a soluble molecule and was secreted outside the cell. The concentration of IL‐12 in the cell supernatant reached 600 pg/mL, and the concentration of GM‐CSF reached 700 pg/mL. Animal experiments showed that the local application of PD‐1v, IL‐2/15, IL‐12, and GM‐CSF can synergistically produce a stronger anti‐tumor immunotherapeutic effect. Our studies further confirmed that the combined application can effectively stimulate the expression of IFN‐γ, TNF, IL‐4, IL‐2, IL‐5, and other cytokines, suggesting that multiple gene combination therapy can effectively enhance immune response, reverse immune tolerance, and inhibit tumor growth. Although the data of receiving combination therapy are encouraging, the limitations of recombinant plasmids in patients' primary tumor cells are still not negligible. Of note, this study is limited to single cell lines, and additional cell lines and comparative studies with viral vectors are needed.

## AUTHOR CONTRIBUTIONS

**Hongxia SU:** Conceptualization (equal); data curation (equal); formal analysis (equal); investigation (equal); methodology (equal); project administration (equal); resources (equal); software (equal); validation (equal); visualization (equal); writing – original draft (equal); writing – review and editing (equal). **Hui Geng:** Writing – review and editing (equal). **Linkang Cai:** Conceptualization (equal). **Minjie Xu:** Data curation (equal). **Wenping Xing:** Formal analysis (lead). **Wei Long:** Methodology (lead). **Biao Liu:** Software (lead). **Yankun Li:** Conceptualization (equal); methodology (equal); writing – review and editing (equal).

## Data Availability

The data that support the findings of this study are available the article.
